# Supplementary material for: Cold stress induces rapid gene-specific changes in the levels of H3K4me3 and H3K27me3 in Arabidopsis thaliana
Source: Front Plant Sci. 2024 Apr 15;15:1390144. doi: 10.3389/fpls.2024.1390144 (PMC11056581; doi:10.3389/fpls.2024.1390144)
Supplement: Supplementary file 1 [file DataSheet_1.docx]

Cold stress induces rapid gene-specific changes in the levels of H3K4me3 and H3K27me3 in *Arabidopsis thaliana*

Supplementary Material


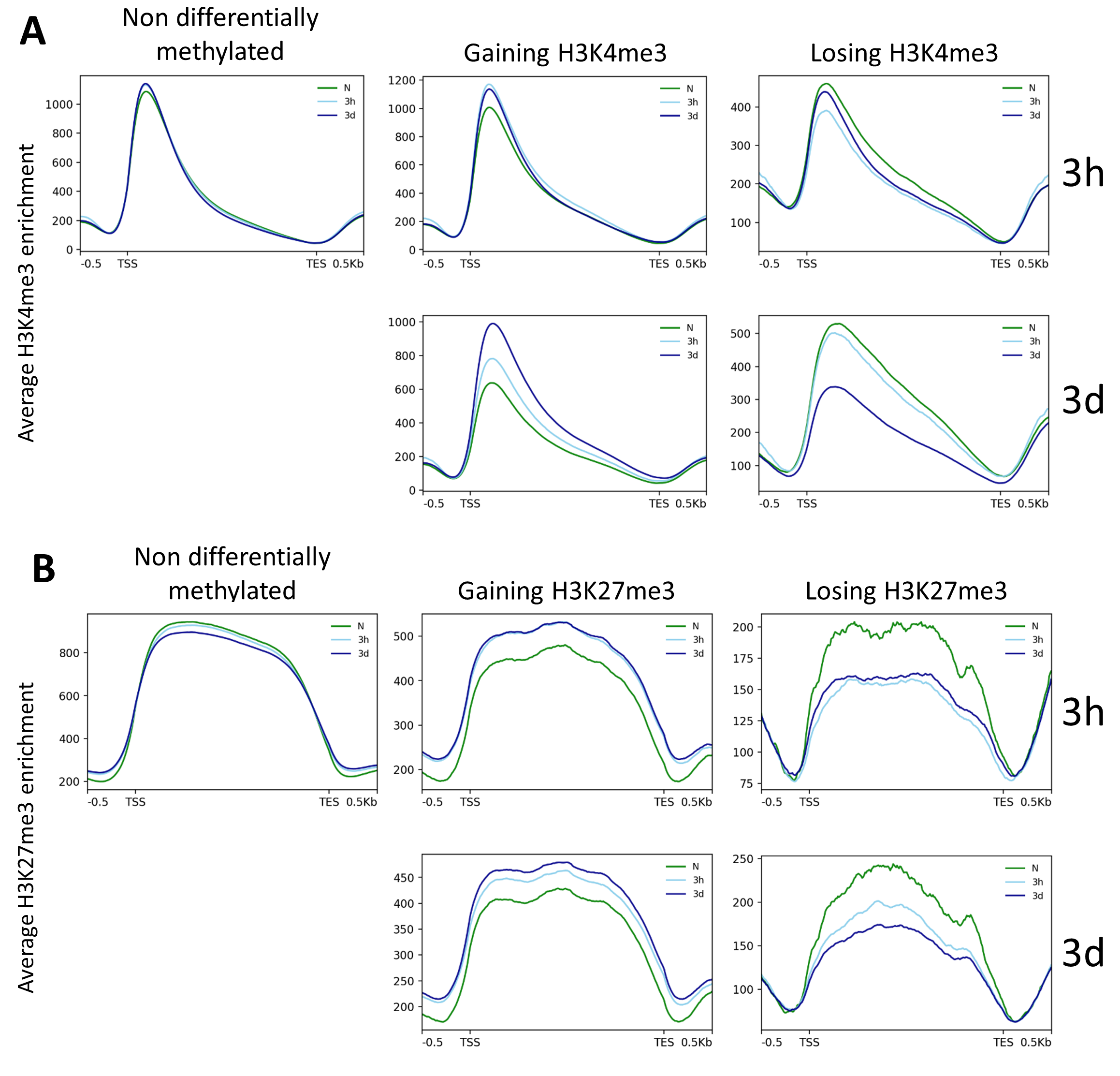


**Sup. Figure 1: Methylation levels on differentially methylated genes.** Metagene plots showing the levels of H3K4me3 **(A)** and H3K27me3 **(B)** on non-differentially methylated genes (left panel), genes gaining (middle panel) or losing the respective mark (right panel), after three hours (3h) or three days (3d) of cold exposure. Differentially methylated genes are genes showing an absolute log2 fold change >= 0.5 of the respective methylation mark on a region spanning from the TSS to the TES.


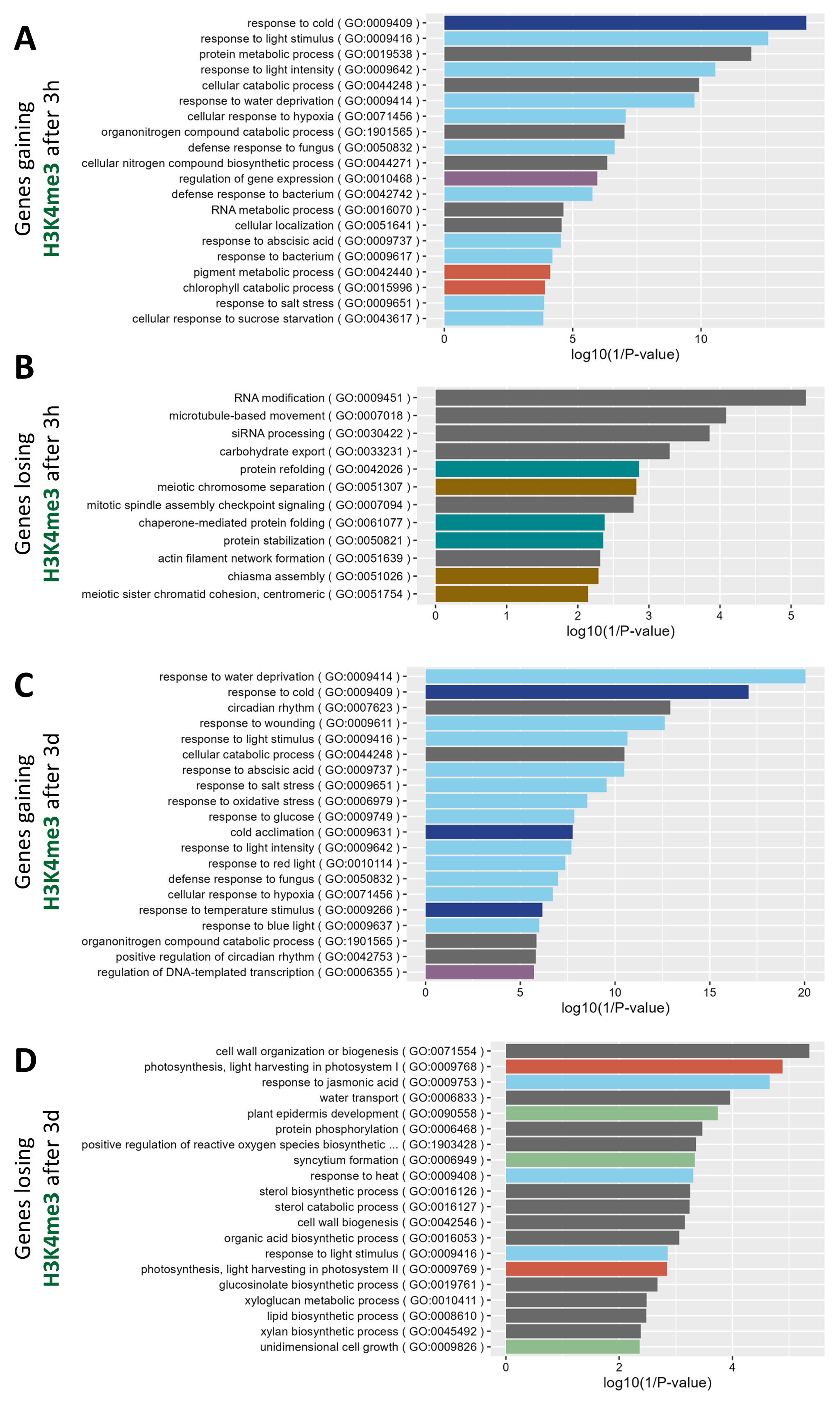


**Sup. Figure 2: Gene ontology (GO) analysis of genes undergoing H3K4me3 differential methylation upon cold exposure.** Enriched biological process GO terms of genes gaining (**(A)** and **(C)**) or losing (**(B)** and **(D)**) H3K4me3 after three hours (**(A)** and **(B)**) or three days (**(C)** and **(D)**) of cold exposure. Colors indicate the broad category of the term: light blue: stress response, dark blue: temperature response, purple: regulation of transcription, orange: photosynthesis, turquoise: protein folding, gold: chromatin, green: development.


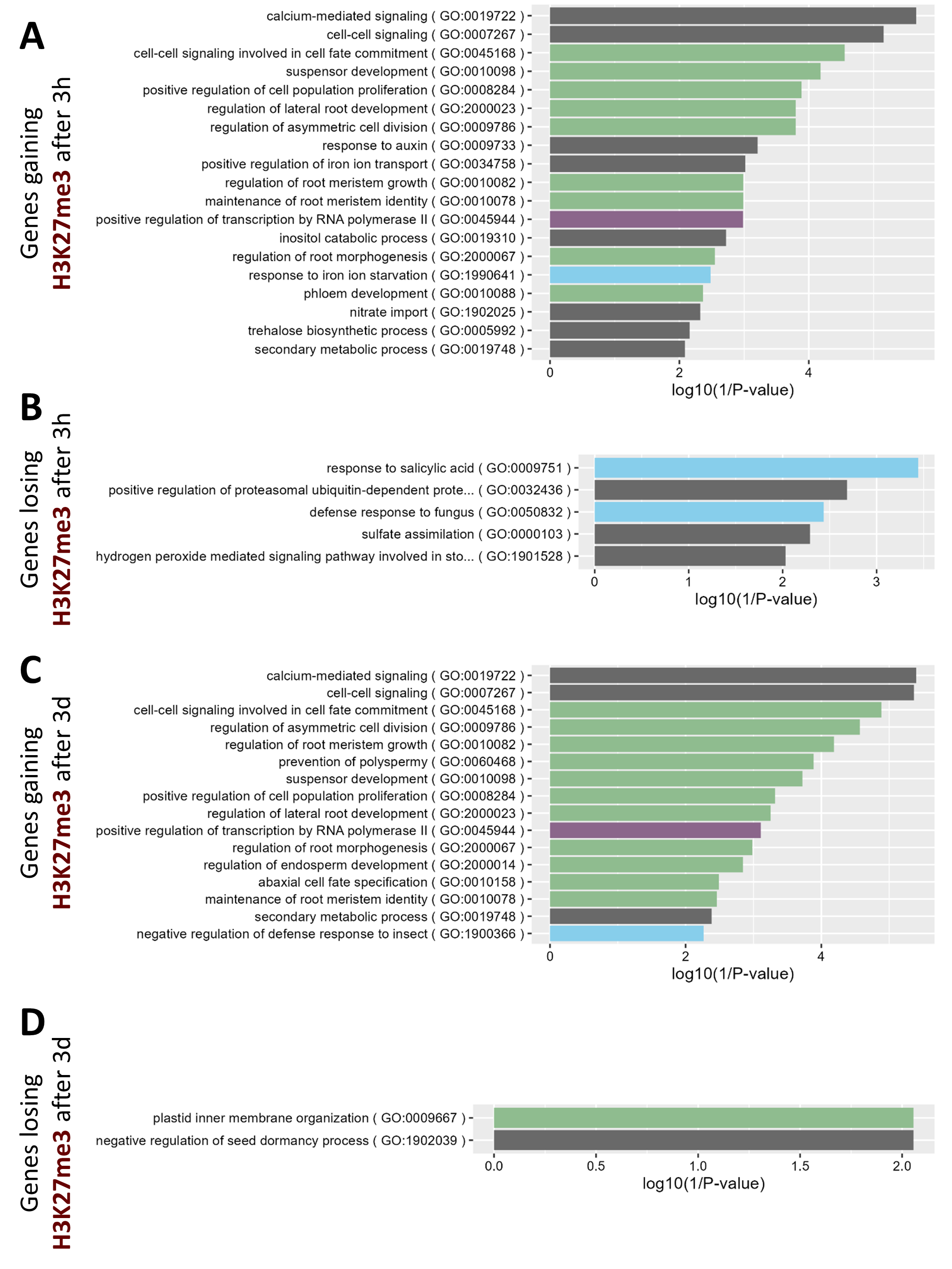


**Sup. Figure 3: Gene ontology (GO) analysis of genes undergoing H3K27me3 differential methylation upon cold exposure.** Enriched biological process GO terms of genes gaining (**(A)** and **(C)**) or losing (**(B)** and **(D)**) H3K27me3 after three hours (**(A)** and **(B)**) or three days (**(C)** and **(D)**) of cold exposure. Colors indicate the broad category of the term: light blue: stress response, purple: regulation of transcription, green: development.


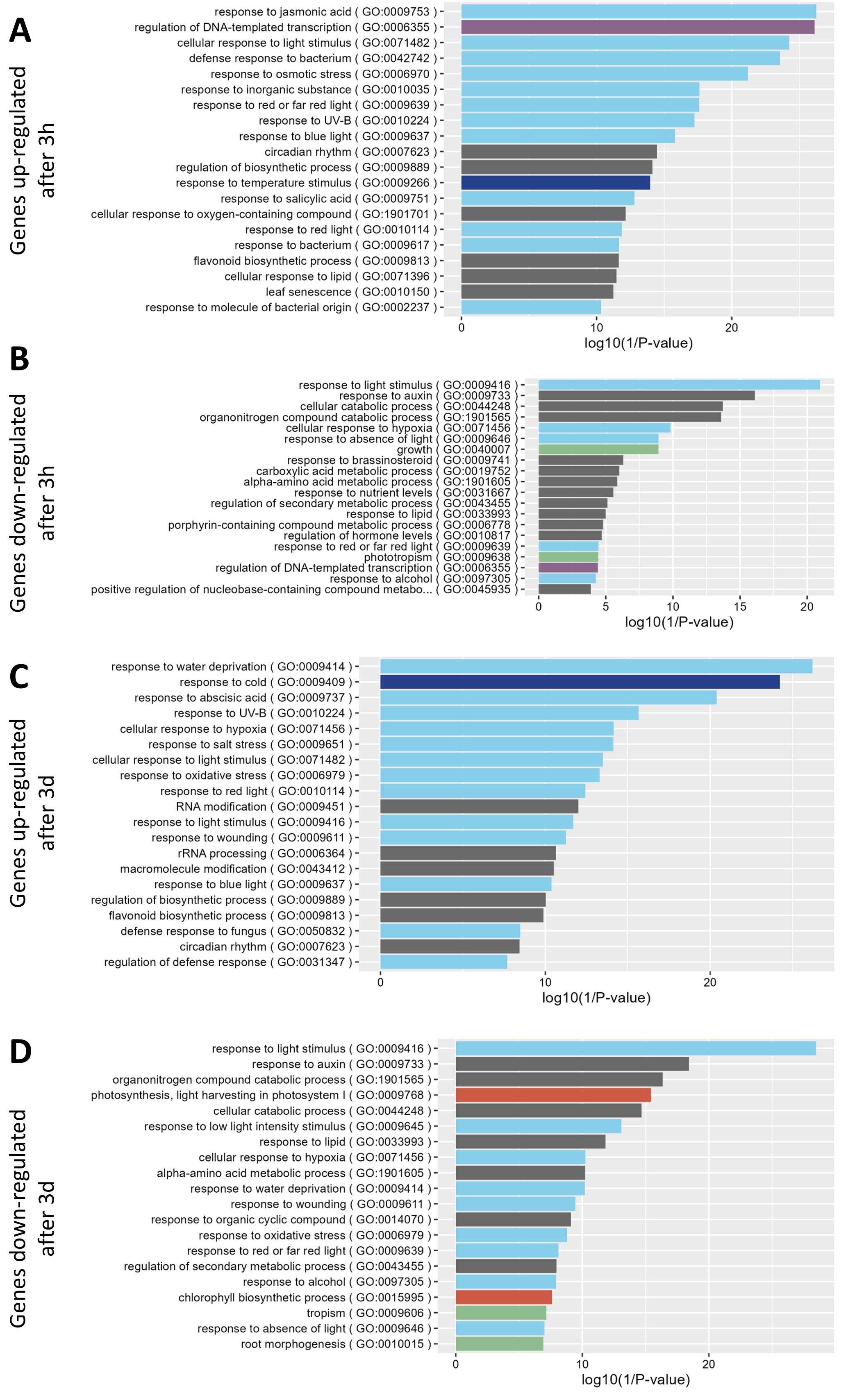


**Sup. Figure 4: Gene ontology (GO) analysis of differentially expressed genes upon cold exposure.** Enriched biological process GO terms of up- (**(A)** and **(C)**) or down-regulated genes (**(B)** and **(D)**) after three hours (**(A)** and **(B)**) or three days (**(C)** and **(D)**) of cold exposure. Colors indicate the broad category of the term: light blue: stress response, dark blue: temperature response, purple: regulation of transcription, orange: photosynthesis, green: development.


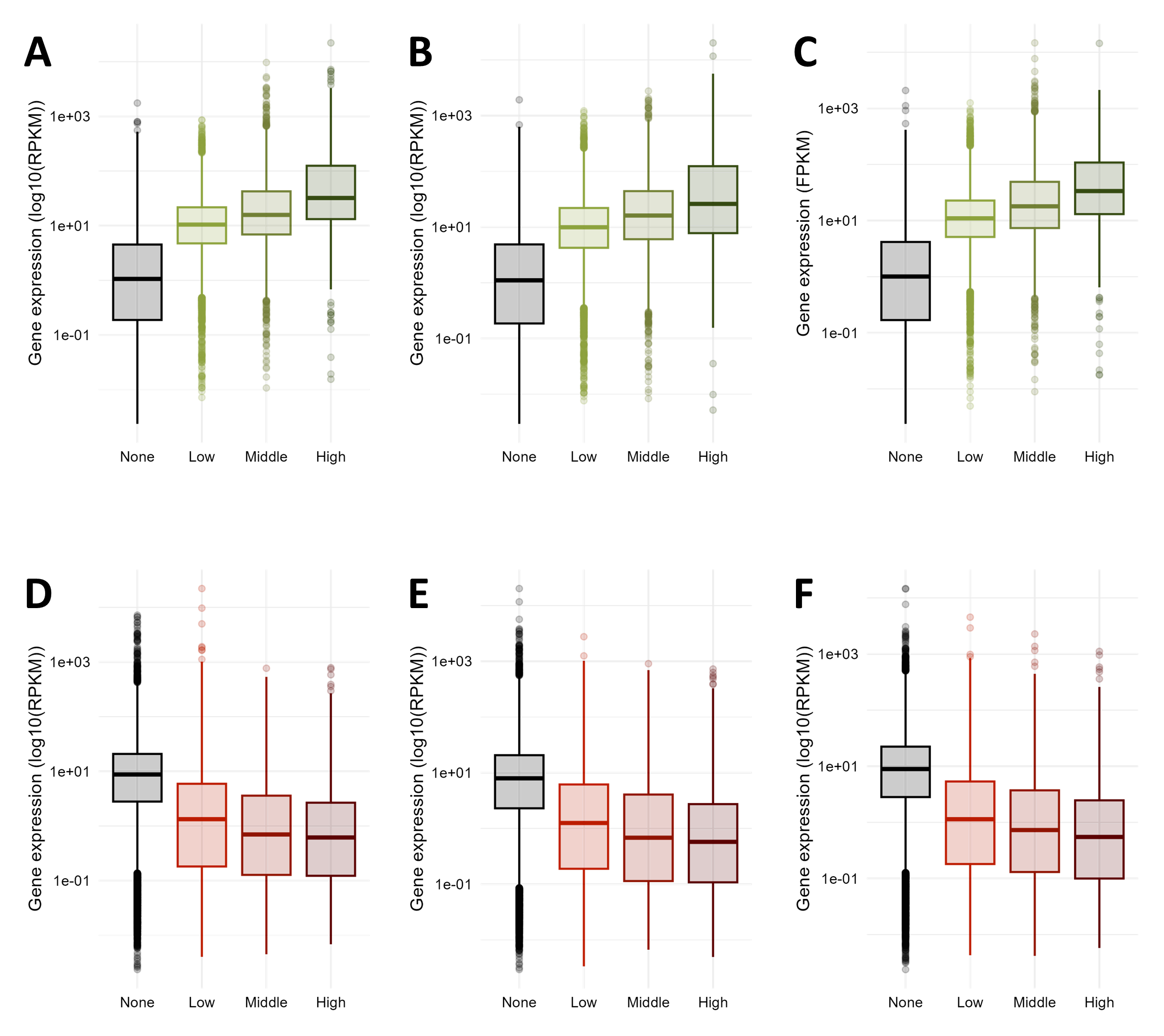


**Sup. Figure 5: H3K4me3 and H3K27me3 levels correlate positively and negatively with gene expression.** **(A)** to **(C)** Box plots showing the distribution of gene expression depending on the levels of H3K4me3, prior to cold exposure **(A)**, after three hours **(B)** or three days **(C)** of cold exposure. Genes were divided in genes displaying no H3K4me3 (none, log10(RPKM) < 1), low (log10(RPKM) <= 1.5), middle (log10(RPKM) <= 2 or high (log10(RPKM) > 2) levels of H3K4me3. **(D)** to **(F)** Box plots showing the distribution of gene expression depending on the levels of H3K27me3, prior to cold exposure **(D)**, after three hours **(E)** or three days **(F)** of cold exposure. Genes were divided in genes displaying no H3K27me3 (none, log10(RPKM) < 1), low (log10(RPKM) <= 1.5), middle (log10(RPKM) <= 2 or high (log10(RPKM) > 2) levels of H3K27me3.


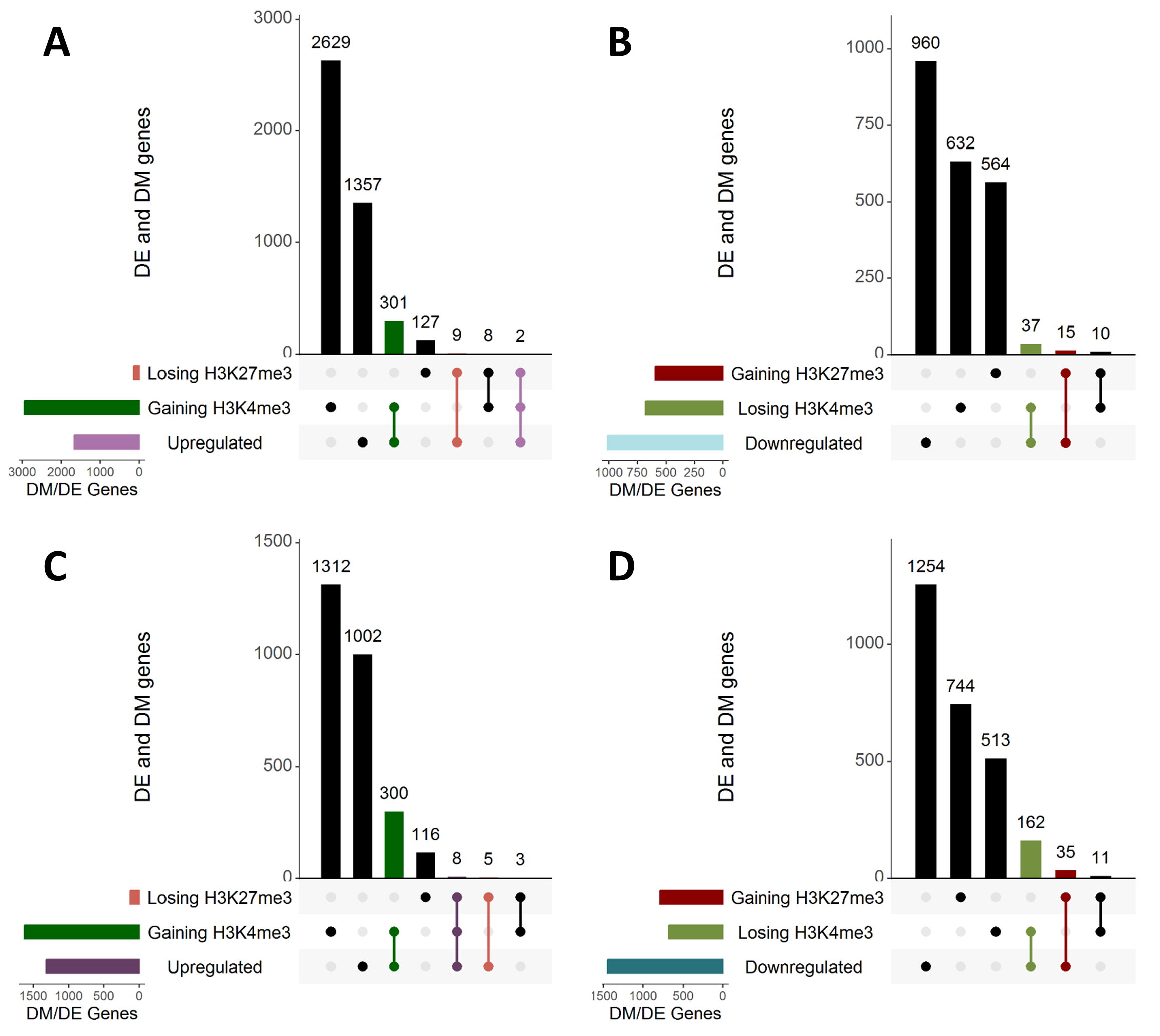


**Sup. Figure 6: Differentially methylated and differentially expressed genes partially overlap.** UpSet plots showing the overlaps between up-regulated genes, genes gaining H3K4me3 or losing H3K27me3 after 3h **(A)** or 3d **(C)** of cold exposure. UpSet plots showing the overlaps between down-regulated genes, genes losing H3K4me3 or gaining H3K27me3 after 3h **(B)** or 3d **(D)** of cold exposure. Intersections of DE genes with H3K4me3 or H3K27me3 DM genes are highlighted in green and red, respectively. Intersection of DE genes with H3K4me3 and H3K27me3 DM genes are highlighted in purple.


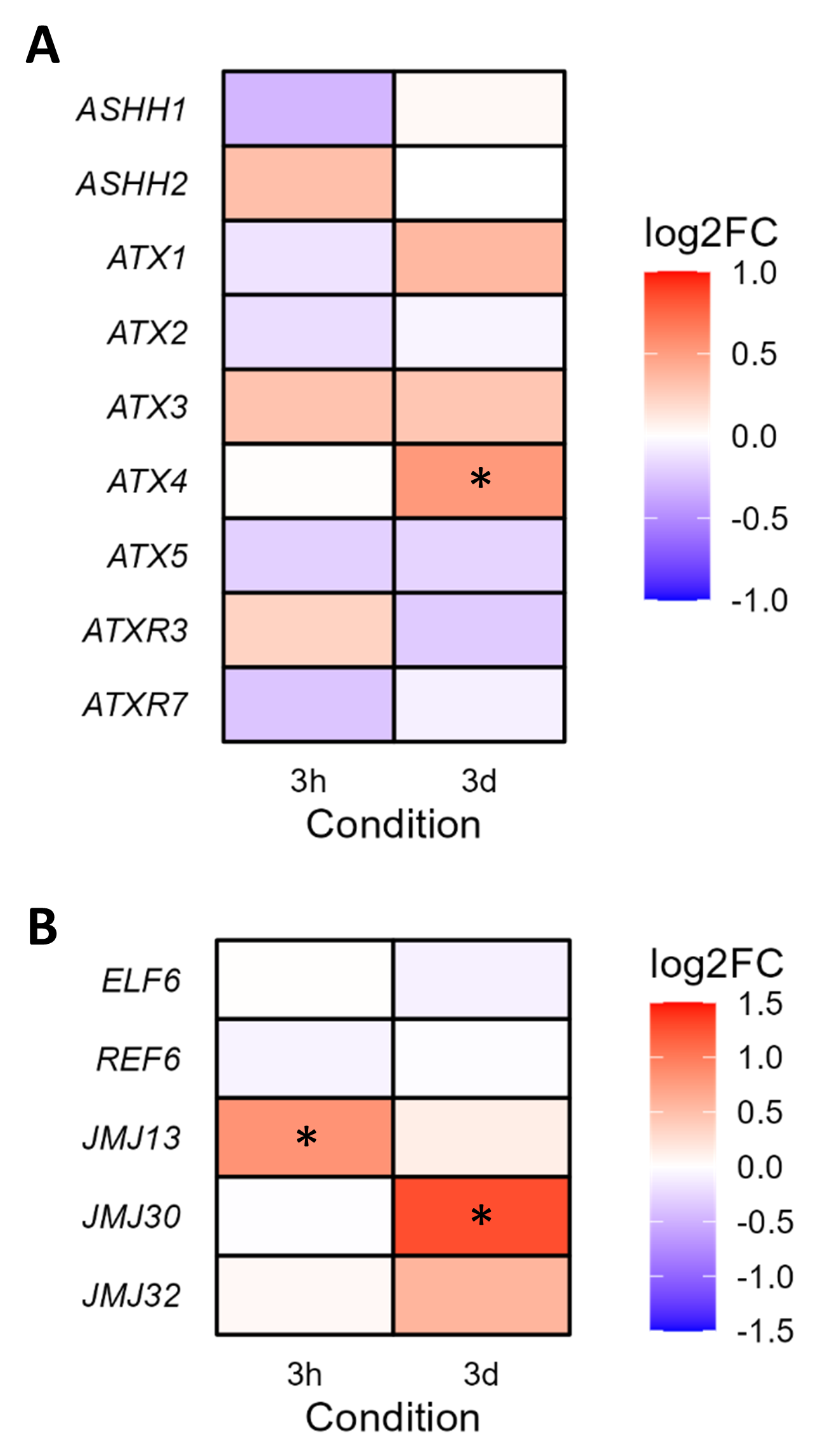


**Sup. Figure 7: Expression changes of genes encoding H3K4me3 methylases (A) or H3K27me3 demethylases (B).** Plants were exposed to cold for either 3h or 3d and the gene expression was analyzed using a RNA-seq. The color indicates the log2 fold change compared to non-treated condition. * indicates p-adj < 0.05
